# Drug-Coated Balloon-Only Strategy for De Novo Coronary Artery Disease: A Meta-analysis of Randomized Clinical Trials

**DOI:** 10.1155/2023/3121601

**Published:** 2023-08-08

**Authors:** Wenyi Zhang, Mingduo Zhang, Jinfan Tian, Min Zhang, Yuan Zhou, Xiantao Song

**Affiliations:** Department of Cardiology, Beijing Anzhen Hospital, Capital Medical University, Beijing Institute of Heart Lung and Blood Vessel Disease, Beijing, China

## Abstract

**Backgrounds:**

Many clinical trials have demonstrated the value of drug-coated balloons (DCB) for in-stent restenosis. However, their role in de novo lesions is not well documented. The aim of this study is to evaluate the safety and efficacy of the DCB-only strategy compared to other percutaneous coronary intervention strategies for de novo coronary lesions.

**Methods:**

The PubMed, Embase, Web of Science, and Cochrane Library Central Register of Controlled Trials (CENTRAL) electronic databases were searched for randomized controlled trials published up to May 6, 2023. The primary outcomes were major adverse cardiac events and late lumen loss.

**Results:**

A total of eighteen trials with 3336 participants were included. Compared with drug-eluting stents, the DCB-only strategy was associated with a similar risk of major adverse cardiac events (risk ratio (RR) = 0.90; 95% confidence interval (CI): 0.59 to 1.37, *P* = 0.631) and a significant decrease in late lumen loss (standardized mean difference (SMD) = −0.29, 95% CI: −0.53 to −0.04, *P* = 0.021). This effect was consistent in subgroup analysis regardless of indication, follow-up time, drug-eluting stent type, and dual antiplatelet therapy duration. However, DCBs were inferior to DESs for minimum lumen diameter and percentage diameter stenosis. The DCB-only strategy showed significantly better outcomes for most endpoints compared to plain-old balloon angioplasty or bare metal stents.

**Conclusions:**

Interventions with a DCB-only strategy are comparable to those of drug-eluting stents and superior to plain-old balloon angioplasty or bare metal stents for the treatment of selected de novo coronary lesions. Additional evidence is still warranted to confirm the value of DCB before widespread clinical utilization can be recommended.

## 1. Introduction

The devices and techniques for percutaneous coronary intervention (PCI) continue to evolve and have already revolutionized the treatment of coronary artery disease. The advent of plain-old balloon angioplasty (POBA) and bare metal stents (BMS) makes it possible for the invasive expansion of stenosed coronary vessels. However, these techniques are limited by complications, such as elastic recoil, abrupt vessel closure, or postprocedure restenosis [1]. Drug-eluting stents (DESs) have considerably reduced the incidence of late stent thrombosis (ST) and clinical outcomes such as myocardial infarction (MI) or target lesion revascularization (TLR) compared to previous devices [2]. However, DES use is still suboptimal in some clinical and anatomic scenarios, such as small coronary vessels, bifurcation lesions, and high risk for bleeding. Moreover, there remains a significant rate of in-stent restenosis (ISR) or ST at follow-up caused by the permanent presence of metal devices [3].

DCBs were first introduced as a treatment for stent-related restenosis and were shown to have promising results. Based on sufficient evidence, DCBs have been recommended as the first-line treatment option for ISR by the European Guidelines [4]. With the advantages of avoiding permanent implants, practitioners have been attempting to use the DCB-only strategy for the treatment of de novo coronary lesions. Results from these studies have been encouraging, especially in select lesion types such as small vessels in which stent therapy was unable to achieve expected results [5]. In recent years, there has been evidence that other clinical situations, such as bifurcation lesions, large coronary vessels, or even complex coronary lesions, may also benefit from DCB-only strategy [6–8].

Although some previous meta-analyses studying the effect of DCBs with default BMS implantation did not seem to produce favorable results, meta-analyses addressing the efficacy and safety of DCB alone (with bailout stenting only) approach were relatively few. Therefore, the present meta-analysis is aimed at summarizing the available evidence and comparing the DCB-only approach with other PCI strategies for the treatment of de novo coronary lesions.

## 2. Methods

### 2.1. Search Strategy

The PubMed, Embase, Web of Science, and Cochrane Library Central Register of Controlled Trials (CENTRAL) electronic databases were searched from inception until May 6, 2023. All published randomized controlled trials (RCTs) that compared the DCB-only approach with other PCI strategies for the treatment of patients with de novo coronary artery disease were identified. A search algorithm was used with a combination of relevant terms. No filters or language restrictions were applied. The detailed search strategy for each database has been provided in Supplementary Table 1. The meta-analysis was performed according to the Preferred Reporting Items for Systematic Reviews and Meta-analysis (PRISMA) guidelines, and the protocol was registered with PROSPERO (CRD42020158856).

### 2.2. Eligibility Criteria

All eligible studies meeting the following inclusion criteria were selected: (1) randomized controlled trials, (2) comparing DCB-only approach (bailout stents were allowed when required) with a control treatment (POBA, BMS, or DES), (3) patients in the study had de novo coronary artery disease, and (4) availability of angiographic or clinical outcome data without follow-up duration restriction. For studies with more than one follow-up period, the longest available angiographic and clinical follow-up results were considered for analysis. Studies that employed routine stents in the DCB group, studies that are not yet finished, and those with incomplete baseline data or follow-up results were excluded.

### 2.3. Primary and Secondary Endpoints

The primary endpoints were major adverse cardiac events (MACEs) and in-segment late lumen loss (LLL). Secondary endpoints included target lesion revascularization (TLR), all-cause death or cardiac death, myocardial infarction (MI), binary restenosis (BR), minimum lumen diameter (MLD), and percent diameter stenosis (DS%).

### 2.4. Data Collection

The search process and data extraction were conducted by two independent investigators (W.Y.Z, M.D.Z). Conflicts were discussed and resolved by consensus. A standardized database (Microsoft Excel) was used to extract details on study information (publication year, design, sample size, and follow-up duration), patient characteristics (age, sex, and comorbidities), PCI devices and strategies, angiographic measures at baseline, and outcomes of interest at follow-up. Qualities of the included studies were assessed by the Cochrane risk of bias assessment tool for RCTs [9].

### 2.5. Statistical Analysis

Statistical analysis was performed using the Stata version 12.0 software (Stata Corp., College Station, Texas, USA). Combined risk ratios (RR) with a 95% confidence interval (CI) and mean differences (MDs) with standard deviations were presented as summary statistics, and results were presented as forest plots. Results were considered statistically significant when *P* values were < 0.05 in two-tailed tests. The heterogeneity among trials was assessed using Cochran's *Q* test and means of the *I*^2^ statistic [10]. *P* values of < 0.10 or *I*^2^ > 50% were considered significant for heterogeneity. We used a mixed-effects model to synthesize data. Both the random-effects model (DerSimonian and Laird) and the fixed-effects model (Mantel-Haenszel) were used to perform the analyses. Considering the differences in the designs, populations, types of interventions, and treatment effects across studies, a random-effects model was given preference for this meta-analysis. Pooled risk estimates were also examined by a fixed-effects model to avoid small studies being overly weighted. Furthermore, it has been recommended that a predictive interval, which reflects the variability of the treatment effects over different settings, should be routinely presented in the random-effects meta-analysis [11]. We have therefore calculated and reported prediction intervals in our meta-analysis.

Publication bias was evaluated using a funnel plot as well as Egger's test [12], and *P* < 0.05 suggested positive evidence of bias. Sensitivity analysis was performed by excluding one trial at a time to assess the contribution of each individual study to the summary statistics. Subgroup analyses between the DCB and DES groups were conducted according to vessel diameter, follow-up duration, clinical diagnosis, DES type, and DAPT period.

## 3. Results

### 3.1. Search Results and Study Characteristics

**Figure 1:**
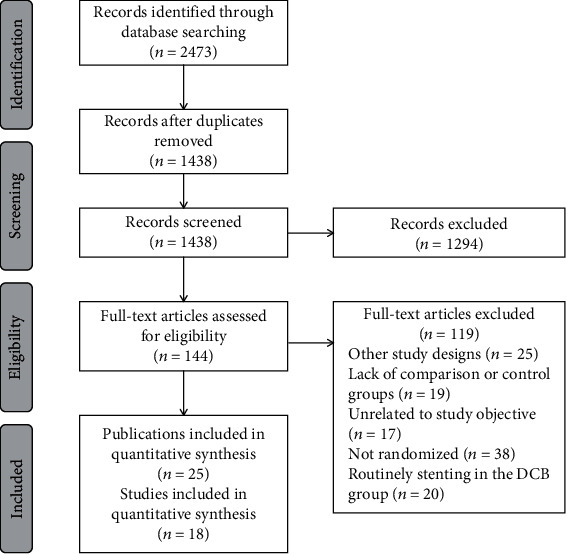
Study selection flow diagram. Abbreviations: DCB: drug-coated balloon.

A total of 2473 articles were identified, of which 18 trials (25 publications, 3336 participants) satisfied the outlined inclusion criteria and were included in the meta-analysis [13–37]. A flow diagram of the search and selection process is shown in Figure 1.  Table 1 shows the characteristics of the included studies. Studies were recruited from different patient populations (small vessels, bifurcations, high bleeding risk, and acute MI). For the control treatments, most trials compared DCB to DES (or DES with a small proportion of BMS) (*n* = 10), five trials compared DCB to POBA [20, 21, 23, 26, 27], and three trials compared DCB to BMS (or BMS with a small proportion of DES) [22, 28, 36]. Supplementary Table 2 shows the baseline patient characteristics in the DCB and control groups. Although there was some variability in the proportion of patients with comorbidities across trials, the baseline characteristics were balanced between the DCB treatment group and the control group.

### 3.2. Primary Endpoints

**Figure 2:**
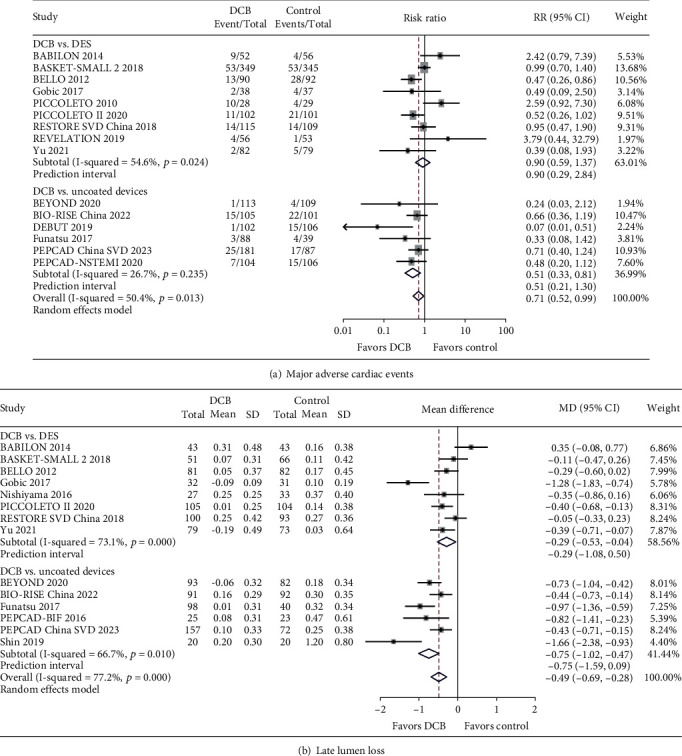
Forest plot of major adverse cardiac events and late lumen loss comparing DCB versus the control treatment. Abbreviations: CI: confidence interval; DCB: drug-coated balloon; DES: drug-eluting stent; MD: mean differences; RR: risk ratio; SD: standard deviations.

With respect to the primary safety endpoint, there were some differences in the definition of MACEs across studies (Table 1). Compared to the DCB-only group, there were no significant differences for MACEs observed in the DES group (RR = 0.90, 95% CI: 0.59 to 1.37, *P* = 0.631), whereas a higher risk of MACEs was found in the BMS or POBA group (RR = 0.51, 95% CI: 0.33 to 0.81, *P* = 0.004) (Figure 2).

Data for LLL were available in 14 trials. The DCB-only group exhibited a significant decrease in LLL compared to DES (SMD = −0.29, 95% CI: -0.53 to -0.04, *P* = 0.021) or uncoated device groups (SMD = −0.75, 95% CI: -1.02 to -0.47, *P* < 0.001) (Figure 2).

The random-effects model was used for the analysis, and similar results were obtained by the fixed-effects model (Supplementary Table 3). The 95% predictive interval for the primary endpoints contained the null effect, indicating that DCBs may exhibit no or an opposite effect compared with the control treatment in all considered settings (Figure 2).

### 3.3. Secondary Endpoints

The differences between the DCB and DES groups were not statistically significant for the risk of TLR (RR = 1.15, 95% CI: 0.56 to 2.34, *P* = 0.705), death or cardiac death (RR = 0.95, 95% CI: 0.61 to 1.48, *P* = 0.825), and MI (RR = 0.80, 95% CI: 0.49 to 1.32, *P* = 0.387). When comparing DCB-only against the BMS/POBA group, DCBs significantly reduced the risks of TLR (RR = 0.42, 95% CI: 0.23 to 0.76, *P* = 0.004), death or cardiac death (RR = 0.39, 95% CI: 0.16 to 0.94, *P* = 0.036), and MI (RR = 0.31, 95% CI: 0.13 to 0.74, *P* = 0.08) (Table 2 and Supplementary Figures 1-3).

For the secondary angiographic outcomes, there was no significant difference between the DCB and DES groups in terms of BR (RR = 1.06, 95% CI: 0.74 to 1.51, *P* = 0.748). However, a statistically significant increase in MLD (SMD = −0.48, 95% CI: -0.67 to -0.29, *P* < 0.001), as well as a significant reduction of DS% (SMD = 0.24, 95% CI: 0.09 to 0.40, *P* = 0.001), was observed in the DES-treated patient group compared with the DCB group. Compared to the POBA or BMS group, DCBs presented a significant reduction in BR (RR = 0.31, 95% CI: 0.22 to 0.45, *P* < 0.001), a significant increase in MLD (SMD = 0.54, 95% CI: 0.31 to 0.76, *P* < 0.001), and a significantly lower DS% (SMD = −0.67, 95% CI: -0.89 to -0.44, *P* < 0.001) (Table 2 and Supplementary Figures 4-6).

We used the random-effects model to perform the above analyses. No significant differences were found between the analytical results of the two effect models (Supplementary Table 3). The between-study heterogeneity for most secondary endpoints was not obvious, and the prediction intervals coincided with the respective CI (Table 2).

### 3.4. Subgroup Analysis

**Figure 3:**
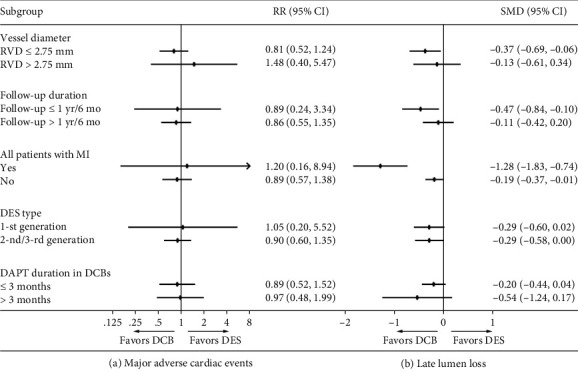
Pooled estimates of effects (95% confidence intervals) for DCB vs. DES in subgroup analyses. Abbreviations: CI: confidence interval; DAPT: dual antiplatelet therapy; DCB: drug-coated balloon; DES: drug-eluting stent; MI: myocardial infarction; RR: risk ratio; RVD: reference vessel diameter.

To further compare the safety and efficacy of DCB-only to DES, data were sorted and analyzed according to vessel diameter, follow-up duration, clinical diagnosis, DES type, and dual antiplatelet therapy (DAPT) duration. In subgroup analysis, no statistically significant difference was detected in MACEs between the DCB and DES groups. However, the advantage of DCB in reducing LLL tended to decrease with the increasing of vessel diameter and follow-up duration (Figure 3).

### 3.5. Bias Assessment and Sensitivity Analysis

The risk of bias assessment in each individual study is presented in Supplementary Figure 7. Overall, most of the studies included were of a relatively high quality and did not reveal significant sources of bias.

Publication bias was assessed by funnel plots for the primary endpoints. The funnel plots were substantially symmetrical according to a visual inspection (Supplementary Figure 8). The absence of bias was also confirmed by Egger's test (*P* = 0.417 for MACEs and *P* = 0.111 for LLL).

Sensitivity analysis was conducted by sequentially excluding one individual study at a time if heterogeneity was identified (*P* < 0.10 or *I*^2^ > 50%) at observed endpoints. Results suggested that no study significantly influenced the overall estimates (Supplementary Figure 9).

## 4. Discussion

DCB has proven highly effective for the treatment of ISR, but its role in de novo lesions is not well documented. The principal findings of our study are as follows: (1) for specific de novo lesions or clinical scenarios (i.e., bifurcation lesions, small-vessel disease, or high bleeding risk), the DCB-only strategy represented a more effective and safer treatment compared to POBA or BMS. (2) There were no significant differences in the primary outcomes between the DCB-only and the DES group; however, DESs were associated with more favorable angiographic endpoints such as MLD and DS%. (3) In subgroup analyses, the DCB-only strategy performed comparably to DES regardless of vessel diameter, follow-up duration, clinical diagnosis, DES type, and DAPT duration, but the advantage of DCB may dwindle as the increase of vessel diameter and follow-up duration.

Although DES has become the main therapy for coronary artery disease, POBA and BMS may also play a part in specific anatomical or clinical settings. The PEPCAD-BIF trial is aimed at exploring the effect of the DCB-only strategy in distal main or side branches of bifurcation lesions. Results showed that compared to POBA, DCBs had a statistically significant reduction in LLL and lower risks of restenosis [26]. The DEBUT randomized controlled trial conducted in patients with elevated bleeding risk demonstrated a higher risk of MACE in the BMS group compared with the DCB group [22]. In the present meta-analysis, studies comparing DCB to BMS or POBA were few, but results were evident. DCBs performed better in most angiographic and clinical outcomes than the BMS/POBA treatment for selected de novo lesions.

Newer generation DES has been shown to be effective and has become the first choice for de novo coronary stenosis. However, some limitations, such as stent thrombosis, stent restenosis, or long-term DAPT, can have an adverse impact on the prognosis. Therefore, researchers are exploring DCB as a promising option for the treatment of de novo coronary artery disease. The DCB followed by routine BMS implantation (DCB+BMS) strategy has been widely investigated as a replacement for DES. However, the results have not been very compelling [38, 39]. Recently, studies focusing on the use of a DCB-only strategy have shown promising results.

The DCB-only strategy was first adopted in small coronary vessels. Although the earlier PICCOLETO trial failed to demonstrate the safety of DCB in small coronary vessels [29], the subsequent BELLO trial comparing the IN.PACT Falcon DCB to the Taxus Libertè DES confirmed the clinical efficacy of the DCB-only strategy in small vessel disease over 3 years of follow-up [19]. Similar results were also reported in the randomized PICCOLETO II and RESTORE SVD China trials [31, 33]. For large de novo coronary vessels, there is growing evidence for the efficacy of DCB as well. A trial conducted by Shin et al. demonstrated that the DCB treatment guided by FFR was safe and effective [40]. Another study showed that DCB for large coronary arteries with diameters > 2.75 mm had a similar risk of MACEs and TLR compared to small vessel lesions, demonstrating a similar efficacy for large and small vessels [41]. Recently, there are also randomized trials that found comparable angiographic and clinical outcomes of the DCB-only group compared with the DES group for treating de novo lesions in large vessels [25, 37].

In addition, the DCB-only strategy also presented a potential advantage in other anatomical or clinical settings. The REVELATION trial showed noninferiority of DCB compared to the second-generation DES regarding clinical and angiographic endpoints in patients with STEMI [34]. Similar results were presented in the PEPCAD-NSTEMI trial for non-ST-segment elevation myocardial infarction [28]. In a retrospective study, the efficacy and safety of the DCB-only strategy in de novo ostial coronary lesions have also been demonstrated [42]. Although there are as yet no randomized trials comparing directly DCB versus DES for chronic total occlusion (CTO) lesions, some cohort studies have reported that the DCB-only strategy is a feasible treatment option in de novo CTO lesions if the result after predilatation is satisfactory [43, 44]. These findings suggest that the DCB-only strategy for the treatment of more complex de novo lesions might be worth exploring.

By summarizing the available evidence, this study demonstrated that DCB showed comparable safety and efficacy with the DES treatment. Given the fact that DES is the mainstay of therapy for de novo lesions, we emphatically compared the safety and efficacy between DCB and DES in subgroup analysis. Although subgroup analyses of trials comparing DCB to DES have shown that the results were stable regardless of vessel diameter, follow-up duration, clinical diagnosis, DES type, and DAPT duration, it should be noted that the effects of DCB in reducing LLL were less evident in some subgroups, such as large coronary vessels and longer follow-up time. Additionally, the DES group appeared to be more favorable than the DCB-only group in terms of angiographic outcomes, showing a significant increase in MLD and a significant reduction in DS%. Accordingly, further randomized controlled trials are still required to demonstrate the long-term benefits of DCB in various types of de novo coronary lesions, especially in de novo lesions of large coronary vessels.

Although there have been meta-analyses investigating the use of the DCB-only strategy in de novo lesions, most of them focused on specific indications, such as small vessels [45–48], large coronary vessels [6], or bifurcation lesions [49]. In the present meta-analysis, we comprehensively evaluated the impact of the DCB-only strategy on angiographic and clinical outcomes in different types of de novo coronary lesions. In addition, we excluded nonrandomized studies to minimize possible selection bias. Some earlier meta-analyses with similar purposes found that the DCB-only strategy was associated with a lower incidence of MI or mortality compared with alternative strategies [50, 51]. However, we found no difference between DCBs and DESs for all the clinical endpoints, including mortality and MI. The results of the angiographic outcomes comparing DCB with DES were also different among the studies. Our meta-analysis, including the most recent trials, did not demonstrate that DCBs were associated with favorable angiographic outcomes such as MLD. Taking different interventional modalities as a whole to compare against DCBs in the previous studies might be partly responsible for the differences. Another recent network meta-analysis suggested that DCB-only was associated with higher LLL than DES in patients with ACS, which was not demonstrated in our research [52]. Although the measure of LLL is broadly favorable to DCB in most studies, it should be interpreted with caution due to the larger acute luminal gain after DES implantation, which will lead to LLL favoring DCB PCI. Therefore, analyzing different angiographic parameters comprehensively should be considered when comparing DCBs with DESs [53].

Even though the safety and efficacy of the DCB-only strategy in different settings of de novo CAD were substantiated by the present study, it is very important to perform careful and extensive lesion preparation before using DCB [54]. Conventional balloons should be routinely used. Noncompliant balloons, scoring balloons, rotablation, or directional atherectomy may also require in specific scenarios to achieve optimal lesion preparation. The lack of appropriate preparation can be associated with worse outcomes.

## 5. Limitations

The comprehensive analysis of different endpoints increased the robustness and credibility of the study conclusions. However, considering the lack of large studies, relatively short follow-up period, and insufficient reports of hard clinical endpoints, such as death and cardiac death, the safety of DCB should not be overestimated at this time. Moreover, some studies excluded patients receiving a bailout stent, making the result more favorable for the DCB group. Finally, heterogeneity may have resulted due to multiple types of comparators used in a single study.

## 6. Conclusions

The DCB-only strategy was comparable to DES and superior to POBA or BMS in primary safety and efficacy endpoints for selected de novo coronary lesions. Further studies are warranted to fully elucidate the long-term benefits of DCB compared to DES in various de novo lesions before the more extensive use of DCB can be recommended.

## Figures and Tables

**Table 1:** Main characteristics of the included trials.

Trial/first author	Year	Indication	No. of patients (*n*)	DCB type	Control group	Follow-up, months	Primary endpoint	Definition of MACE	Bailout stenting (%)
BABILON [13]	2014	Bifurcation lesions	108	SeQuent Please	2nd-DES/BMS	9 (angiographic) 24 (clinical)	LLL	Death, MI, TLR	7.8

BASKET-SMALL 2 [14–16]	2018	Small vessel disease	758	SeQuent Please	2nd-gen DES	6 (angiographic) 36 (clinical)	MACE	Cardiac death, MI, TVR	5.1

BELLO [17–19]	2012	Small vessel disease	182	IN.PACT Falcon	1st-gen DES	6 (angiographic) 36 (clinical)	LLL, MACE	Death, MI, TVR	20.2

BEYOND [20]	2020	Bifurcation lesions	222	Bingo	POBA	9 (angiographic) 9 (clinical)	TLS	Death, MI, stroke, TVR	0

BIO-RISE CHINA [21]	2022	Small vessel disease	212	Biolimus A9 (BA9)	POBA	9 (angiographic) 12 (clinical)	LLL	Death, MI, revascularization	2.8

DEBUT [22]	2019	High bleeding risk	208	SeQuent Please	BMS	9 (clinical)	MACE	Cardiac death, MI, TLR	2.0

Funatsu et al. [23]	2017	Small vessel disease	133	SeQuent Please	POBA	6 (angiographic) 6 (clinical)	TVF	Cardiac death, MI, TVR	2.9

Gobić et al. [24]	2017	De novo lesions (STEMI)	78	SeQuent Please	2nd-gen DES	6 (angiographic) 6 (clinical)	MACE, LLL	Cardiac death, MI, TLR, thrombosis	7.3

Nishiyama et al. [25]	2016	De novo lesions	60	SeQuent Please	2nd-gen DES	8 (angiographic) 8 (clinical)	TLR	NR	10.0

PEPCAD-BIF [26]	2016	Bifurcation lesions	64	SeQuent Please	POBA	9 (angiographic) 9 (clinical)	LLL	NR	0

PEPCAD China SVD [27]	2023	Small vessel disease	270	SeQuent Please	POBA	9 (angiographic) 12 (clinical)	LLL	Death, MI, revascularization	2.1

PEPCAD-NSTEMI [28]	2020	De novo lesions (NSTEMI)	210	SeQuent Please	BMS/2nd-DES	9 (clinical)	TLF	Death, MI, stroke, revascularization	14.6

PICCOLETO [29]	2010	Small vessel disease	60	Dior	1st-gen DES	6 (angiographic) 9 (clinical)	DS%	Death, MI, TLR	34.5

PICCOLETO II [30, 31]	2020	Small vessel disease	232	Elutax SV/Emperor	2nd-gen DES	6 (angiographic) 36 (clinical)	LLL	Cardiac death, MI, TLR	6.7

RESTORE SVD China [32, 33]	2018	Small vessel disease	230	Restore	2nd-gen DES	9 (angiographic) 24 (clinical)	DS%	Cardiac death, MI, TLR	5.2

REVELATION [34, 35]	2019	De novo lesions (STEMI)	120	Pantera Lux	2nd-gen DES	9 (angiographic) 24 (clinical)	FFR	Cardiac death, MI, TLR	18.0

Shin et al. [36]	2019	High bleeding risk	40	SeQuent Please	BMS	9 (angiographic) 12 (clinical)	LLL	NR	0

Yu et al. [37]	2021	De novo lesions	170	SeQuent Please	2nd-gen DES	9 (angiographic) 12 (clinical)	LLL MACE	Cardiac death, MI, TLR	2.4

Abbreviations: BMS: bare metal stent; DCB: drug-coated balloon; DES: drug-eluting stent; DS%: percentage diameter stenosis; FFR: fractional flow reserve; MACE: major adverse cardiovascular events; LLL: late lumen loss; MI: myocardial infarction; NR: not reported; NSTEMI: non-ST-segment elevation; POBA: plain-old balloon angioplasty; STEMI: ST-segment elevated myocardial infarction; TLF: target lesion failure; TLR: target lesion revascularization; TLS: target lesion stenosis; TVF: target vessel failure; TVR: target vessel revascularization.

**Table 2:** Pooled estimates of effects (95% confidence intervals) for DCB vs. control treatment for secondary endpoints.

Secondary endpoints	DCB vs. DES					DCB vs. uncoated devices				
	*N*	Pooled estimate (95% CI)	Prediction interval	*P*	*I* ^2^	*N*	Pooled estimate (95% CI)	Prediction interval	*P*	*I* ^2^
*Clinical endpoints*										
TLR	8	1.15 (0.56, 2.34)	(0.17, 7.76)	0.705	51.1%	8	0.42 (0.23, 0.76)	(0.23, 0.76)	0.004	0.0%
Death/cardiac death	10	0.95 (0.61, 1.48)	(0.61, 1.48)	0.825	0.0%	8	0.39 (0.16, 0.94)	(0.16, 0.94)	0.036	0.0%
MI	8	0.80 (0.49, 1.32)	(0.49, 1.32)	0.387	0.0%	8	0.31 (0.13, 0.74)	(0.13, 0.74)	0.008	0.0%
*Angiographic endpoints*										
Binary restenosis	6	1.06 (0.74, 1.51)	(0.74, 1.51)	0.748	0.0%	5	0.31 (0.22, 0.45)	(0.22, 0.45)	<0.001	0.0%
MLD	10	-0.48 (-0.67, -0.29)	(-1.05, 0.10)	<0.001	59.7%	6	0.54 (0.31, 0.76)	(-0.09, 1.17)	<0.001	52.7%
DS%	7	0.24 (0.09, 0.40)	(-0.06, 0.55)	0.001	19.1%	6	-0.67 (-0.89, -0.44)	(-1.28, -0.05)	<0.001	51.1%

*N*: number of eligible studies included. Abbreviations: CI: confidence interval; DCB: drug-coated balloon; DES: drug-eluting stents; DS%: percent diameter stenosis; MI: myocardial infarction; MLD: minimum lumen diameter; TLR: target lesion revascularization.

## Data Availability

Data are available from the corresponding author on request.

## Supplementary Materials

Supplementary Materials Supplementary 1. Table S1: full search strategy and search terms. Supplementary 2. Table S2: patient and disease characteristics in eligible studies. Supplementary 3. Table S3: summary effect sizes for outcomes with DCB vs. the control treatment. Supplementary 4. Figure S1: forest plot of risk ratios for target lesion revascularization associated with DCB vs. the control treatment. Supplementary 5. Figure S2: forest plot of risk ratios for death or cardiac death associated with DCB vs. the control treatment. Supplementary 6. Figure S3: forest plot of risk ratios for myocardial infarction associated with DCB vs. the control treatment. Supplementary 7. Figure S4: forest plot of risk ratios for binary restenosis associated with DCB vs. the control treatment. Supplementary 8. Figure S5: forest plot of mean differences for minimal lumen diameter associated with DCB vs. the control treatment. Supplementary 9. Figure S6: forest plot of mean differences for percentage diameter stenosis associated with DCB vs. the control treatment. Supplementary 10. Figure S7: bias assessment of the included studies according to the Cochrane Collaboration. Supplementary 11. Figure S8: funnel plot for publication bias of studies included in the meta-analysis. Supplementary 12. Figure S9: sensitivity analysis.
