# Supplementary material for: Drug-Coated Balloon-Only Strategy for De Novo Coronary Artery Disease: A Meta-analysis of Randomized Clinical Trials
Source: Cardiovasc Ther. 2023 Aug 8;2023:3121601. doi: 10.1155/2023/3121601 (PMC10427238; doi:10.1155/2023/3121601)
Supplement: Supplementary Materials — Supplementary 1. Table S1: full search strategy and search terms. Supplementary 2. Table S2: patient and disease characteristics in eligible studies. Supplementary 3. Table S3: summary effect sizes for outcomes with DCB vs. the control treatment. Supplementary 4. Figure S1: forest plot of risk ratios for target lesion revascularization associated with DCB vs. the control treatment. Supplementary 5. Figure S2: forest plot of risk ratios for death or cardiac death associated with DCB vs. the control treatment. Supplementary 6. Figure S3: forest plot of risk ratios for myocardial infarction associated with DCB vs. the control treatment. Supplementary 7. Figure S4: forest plot of risk ratios for binary restenosis associated with DCB vs. the control treatment. Supplementary 8. Figure S5: forest plot of mean differences for minimal lumen diameter associated with DCB vs. the control treatment. Supplementary 9. Figure S6: forest plot of mean differences for percentage diameter stenosis associated with DCB vs. the control treatment. Supplementary 10. Figure S7: bias assessment of the included studies according to the Cochrane Collaboration. Supplementary 11. Figure S8: funnel plot for publication bias of studies included in the meta-analysis. Supplementary 12. Figure S9: sensitivity analysis. [file 3121601.f1.docx]

**SUPPLEMENTARY MATERIAL**

**Drug-coated balloon-only strategy for de novo coronary artery disease: a meta-analysis of randomized clinical trials**

**Online-only Data Supplement**

| **Table S1** | Full search strategy and search terms | 2 |
| --- | --- | --- |
| **Table S2** | Patient and disease characteristics in eligible studies | 4 |
| **Table S3** | Summary effect sizes for outcomes with DCB vs. the control treatment | 6 |
| **Figure S1** | Forest plot of risk ratios for target lesion revascularization associated with DCB vs. the control treatment | 7 |
| **Figure S2** | Forest plot of risk ratios for death or cardiac death associated with DCB vs. the control treatment | 8 |
| **Figure S3** | Forest plot of risk ratios for myocardial infarction associated with DCB vs. the control treatment | 9 |
| **Figure S4** | Forest plot of risk ratios for binary restenosis associated with DCB vs. the control treatment | 10 |
| **Figure S5** | Forest plot of mean differences for minimal lumen diameter associated with DCB vs. the control treatment | 11 |
| **Figure S6** | Forest plot of mean differences for diameter stenosis associated with DCB vs. the control treatment | 12 |
| **Figure S7** | Bias assessment of the included studies according to the Cochrane Collaboration | 13 |
| **Figure S8** | Funnel plot for publication bias of studies included in the meta-analysis | 14 |
| **Figure S9** | Sensitivity analysis | 15 |

| **Supplementary Table 1. Full search strategy and search terms** | | |
| --- | --- | --- |
| Database | Search strategy | Results |
| PubMed | (((drug coated balloon OR drug eluting balloon OR paclitaxel eluting balloon OR paclitaxel coated balloon OR sirolimus coated balloon OR sirolimus eluting balloon OR DCB OR PCB OR SCB))) [All Fields] AND ((*de novo* vessel OR *de novo* lesion OR *de novo* narrowing OR *de novo* coronary lesion OR *de novo* coronary artery lesion OR *de novo* coronary stenosis OR *de novo* coronary vessel OR native vessel OR native lesion OR native narrowing OR native coronary lesion OR native coronary artery lesion OR native coronary stenosis OR native coronary vessel OR *de novo* coronary artery disease OR *de novo* native coronary artery lesion OR *de novo* native coronary stenosis OR native coronary artery *de novo* lesion)) [All Fields] | 918 |
| Embase | 1. (drug coated balloon or drug eluting balloon or paclitaxel eluting balloon or paclitaxel coated balloon or sirolimus coated balloon or sirolimus eluting balloon or DCB or PCB or SCB).mp. [mp=title, abstract, full text, caption text]  2. (coronary artery disease or coronary heart disease or coronary artery lesion or coronary lesion or coronary stenosis).mp. [mp=title, abstract, full text, caption text]  3. (*de novo* or native).mp. [mp=title, abstract, full text, caption text]  4. 1 and 2 and 3 | 302 |
| Cochrane Library | (((drug coated balloon OR drug eluting balloon OR paclitaxel eluting balloon OR paclitaxel coated balloon OR sirolimus coated balloon OR sirolimus eluting balloon OR DCB OR PCB OR SCB))) [Title Abstract Keyword] AND ((*de novo* vessel OR *de novo* lesion OR *de novo* narrowing OR *de novo* coronary lesion OR *de novo* coronary artery lesion OR *de novo* coronary stenosis OR *de novo* coronary vessel OR native vessel OR native lesion OR native narrowing OR native coronary lesion OR native coronary artery lesion OR native coronary stenosis OR native coronary vessel OR *de novo* coronary artery disease OR *de novo* native coronary artery lesion OR *de novo* native coronary stenosis OR native coronary artery *de novo* lesion)) [Title Abstract Keyword] | 427 |
| Web of Science | # 1 TS=(drug coated balloon OR drug eluting balloon OR paclitaxel eluting balloon OR paclitaxel coated balloon OR sirolimus coated balloon OR sirolimus eluting balloon OR DCB OR PCB OR SCB)  # 2 TS=(*de novo* vessel OR *de novo* lesion OR *de novo* narrowing OR *de novo* coronary lesion OR *de novo* coronary artery lesion OR *de novo* coronary stenosis OR *de novo* coronary vessel OR native vessel OR native lesion OR native narrowing OR native coronary lesion OR native coronary artery lesion OR native coronary stenosis OR native coronary vessel OR *de novo* coronary artery disease OR *de novo* native coronary artery lesion OR *de novo* native coronary stenosis OR native coronary artery *de novo* lesion)  # 3 #2 AND #1 | 826 |

Search was conducted to identify articles up to May 6, 2023

| **Supplementary Table 2. Patient and disease characteristics in eligible studies** | | | | | | | | | | | | |
| --- | --- | --- | --- | --- | --- | --- | --- | --- | --- | --- | --- | --- |
| Trial | Interventions  N | RVD, mm  mean ± SD | Mean age  mean ± SD | Men  N, (%) | DM  N, (%) | Dyslipidemia  N, (%) | Smoking  N, (%) | Prior MI  N, (%) | ACS  N, (%) | Endpoints | DAPT, months |  |
| BABILON 2014 | DCB (52)  DES (56) | 3.11 ± 0.52  3.02 ± 0.41 | 63.9 ± 11.3  65.6 ± 11.1 | 33 (63.5)  37 (66.1) | 14 (26.9)  20 (35.7) | 36 (69.2)  33 (58.9) | 25 (48.1)  29 (51.8) | 15 (28.8)  12 (21.4) | 23 (44.2)  24 (42.9) | LLL, MACE | 3  12 |  |
| BASKET-SMALL 2 2018 | DCB (382)  DES (376) | NR | 67.2 ± 10.3  68.4 ± 10.3 | 295 (77)  262 (70) | 122 (32)  130 (35) | 262 (69)  259 (70) | 82 (22)  72 (20) | 160 (42)  133 (35) | 112 (30)  102 (27) | MACE | 3  6 |  |
| BELLO 2012 | DCB (90)  DES (92) | 2.15± 0.27*  2.26 ± 0.24 | 64.8 ± 8.5  66.4 ± 9.0 | 72 (80)  71 (77.2) | 39 (43.3)  35 (38) | 71 (78.9)  73 (79.3) | 15 (16.7)  10 (10.9) | 46 (51.1)  33 (35.9) | 22 (24.4)  20 (21.7) | LLL, TLR, MACE, BR | 1  12 |  |
| BEYOND 2020 | DCB (113)  POBA (109) | 2.15 ± 0.33  2.10 ± 0.29 | 59.9 ± 10.1  61.8 ± 9.4 | 90 (79.7)  71 (65.1) | 34 (30.1)  38 (34.9) | 24 (21.2)  28 (25.7) | 63 (55.8)  56 (51.4) | NR | NR | DS%, LLL, MACCE | 12 |  |
| BIO-RISE CHINA 2022 | DCB (105)  POBA (101) | 1.99 ± 0.31  2.05 ± 0.31 | 61.3 ± 8.8  61.6 ± 8.1 | 76 (72.4)  77 (66.3) | 36 (34.3)  35 (34.7) | 18 (17.1)  18 (17.8) | 47 (44.8)  43 (42.6) | 21 (20.0)  27 (26.7) | 63 (60.0)  62 (61.4) | LLL | 3 |  |
| DEBUT 2019 | DCB (102)  BMS (106) | NR | 77.6 ± 8.4  76.2 ± 8.5 | 63 (62)  68 (64) | 27 (26)  52 (49) | 80 (78)  89 (84) | 34 (34)  36 (33) | 36 (33)  20 (19) | 47 (46)  49 (46) | MACE, TLR | 1 |  |
| Funatsu 2017 | DCB (92)  POBA (41) | 2.04 ± 0.39  1.99 ± 0.28 | 68 ± 10  69 ± 11 | 72 (78)  28 (68) | 44 (48)  13 (32) | 68 (74)  36 (88) | NR | NR | NR | TVF, TLR, LLL | ≥ 3 |  |
| Gobić 2017 | DCB (38)  DES (37) | NR | 56.6 ± 13.2  54.3 ± 10.6 | 27 (71.1)  27 (73.0) | 2 (5.3)  4 (10.8) | 4 (10.5)  7 (18.9) | 16 (42.1)  21 (56.8) | NR | 41 (100)  37 (100) | MACE, LLL, BR | 12 |  |
| Nishiyama 2016 | DCB (30)  DES (30) | 2.88 ± 0.57  2.72 ± 0.64 | 67.3 ± 11.1  70.6 ± 9.0 | 20 (66.7)  24 (80.0) | 12 (40.0)  13 (43.3) | 24 (80.0)  23 (76.7) | 17 (56.7)  19 (63.3) | NR | NR | TLR, LLL, DS% | 8 |  |
| PEPCAD-BIF 2016 | DCB (32)  POBA (32) | 2.38 ± 0.38  2.41 ± 0.39 | 66 ± 12  69 ± 10 | 24 (75.0)  23 (71.9) | 11 (34.4)  12 (37.5) | NR | 6 (18.8)  10 (31.0) | 4 (12.5)  8 (25.0) | 9 (28.1)  6 (18.8) | LLL, TLR, MI | 1  12 |  |
| PEPCAD China SVD 2023 | DCB (181)  POBA (87) | 2.03 ± 0.33  2.09 ± 0.36 | 63.8 ± 8.9  63.3 ± 8.4 | 134 (74.0)  62 (71.3) | 58 (32.0)  34 (39.1) | 44 (24.3)  17 (18.5) | 47 (26.1)  21 (24.1) | 31 (17.1)  9 (10.3) | 116 (64.1)  59 (67.8) | LLL, TLR, MACE, thrombosis, TLF | 12 |  |
| PEPCAD-NSTEMI  2020 | DCB (104)  BMS (106) | NR | 66.0 ± 11.4  67.0 ± 13.1 | 69 (66.3)  72 (67.9) | 28 (26.9) 38 (35.8) | 52 (50.0)  48 (45.3) | 35 (33.7)  43 (40.6) | 20 (19.2)  17 (16.0) | 104 (100.0)  106 (100.0) | TLF, MACE | NR |  |
| PICCOLETO 2010 | DCB (28)  DES (29) | 2.45 ± 0.28  2.36 ± 0.25 | 68 ± 9  67 ± 10 | 22 (78.6)  22 (75.9) | 13 (37.9)  11 (46.4) | 17 (60.7)  13 (54.2) | NR | 5 (17.9)  6 (20.7) | 15 (53.6)  16 (55.2) | DS%, BR, MACE | 1  12 |  |
| PICCOLETO II 2020 | DCB (118)  DES (114) | 2.23 ± 0.4  2.18 ± 0.4 | 64 (48-80)  66 (50-82) | 83 (70.3)  87 (76.9) | 45 (38.0)  40 (35.4) | 72 (61)  63 (55) | 23 (19.5)  19 (16.7) | 45 (38)  34 (30) | 37 (31.4)  32 (21.1) | LLL, MLD, BR, MACE | 1  6 |  |
| RESTORE SVD China 2018 | DCB (116)  DES (114) | 2.42 ± 0.15  2.42 ± 0.18 | 60.1 ± 10.5  60.5 ± 10.8 | 66.4 (77)  77.2 (88) | 39.7 (46)  42.1 (48) | 52.6 (61)  48.2 (55) | 29.3 (34)  31.6 (36) | 22.4 (26)  24.6 (28) | 69.0 (80)  71.1 (81) | DS%, LLL, BR, TLF | ≥ 6 |  |
| REVELATION 2019 | DCB (60)  DES (60) | NR | 57.4 ± 9.2  57.3 ± 8.3 | 52 (87)  52 (87) | 8 (13)  4 (7) | 10 (17)  8 (13) | 28 (47)  24 (40) | NR | 60 (100)  60 (100) | FFR, MACE | 12 |  |
| Shin 2019 | DCB (20)  BMS (20) | 3.0 ± 0.4  3.2 ± 0.3 | 57.5 ± 9.2  61.6 ± 9.5 | 14 (70.0)  15 (75.0) | 7 (35.0)  5 (25.0) | 10 (50.0)  10 (50.0) | 8 (40.0)  5 (25.0) | 1 (5.0)  0 | 6 (30)  8 (40) | LLL, CD, MI, TLR | 1 |  |
| Yu 2021 | DCB (84)  DES (79) | 2.77(2.50,3.25)*  3.01(2.65,3.39) | 62.6 ± 8.8  64.0± 10.5 | 62 (73.8)  56 (70.9) | 16 (19.0)  23 (29.1) | 52 (61.9)  39 (49.4) | 46 (54.8)  42 (53.2) | NR | 18 (21.5)  13 (16.5) | LLL, MLD, DS%  MACE | 1-12  12 |  |
| Abbreviations: ACS, Acute Coronary Syndrome; BMS, Bare Metal Stent; BR, Binary Restenosis; CD, Cardiac Death; DAPT, Dual Anti-Platelet Therapy; DCB, Drug-Coated Balloon, DES, Drug-Eluting Stent; DM, Diabetes Mellitus; DS%, Percentage Diameter Stenosis; FFR, Fractional Flow Reserve; LLL, Late Lumen Loss; MACCE, Major Adverse Cardiac and Cerebral Events; MACE, Major Adverse Cardiovascular Events; MI, Myocardial Infarction; MLD, Minimal Luminal Diameter; NR, Not Reported; POBA, Plain Old Balloon Angioplasty; RVD, Reference Vessel Diameter; TLF, Target Lesion Failure; TLR, Target Lesion Revascularization; TVF, Target Vessel Failure.  Values are mean ± SD or median (interquartile range) or n (% of total)  * Statistically significant value (P < 0.05). | | | | | | | | | | | | |

**Supplementary Table 3. Summary effect sizes for outcomes with DCB vs. the control treatment**

| Endpoints | DCB vs. DES | | | | | DCB vs. uncoated devices | | | | |
| --- | --- | --- | --- | --- | --- | --- | --- | --- | --- | --- |
|  | N | RE Effect Sizes  (95% CI) | P | FE Effect Sizes  (95% CI) | P | N | RE Effect Sizes  (95% CI) | P | FE Effect Sizes  (95% CI) | P |
| **Clinical Endpoints** |  |  |  |  |  |  |  |  |  |  |
| MACEs | 9 | 0.90 (0.59, 1.37) | 0.631 | 0.87 (0.69, 1.10) | 0.241 | 6 | 0.51 (0.33, 0.81) | 0.004 | 0.56 (0.40, 0.79) | 0.001 |
| TLR | 8 | 1.15 (0.56, 2.34) | 0.705 | 1.00 (0.64, 1.56) | 0.988 | 8 | 0.42 (0.23, 0.76) | 0.004 | 0.42 (0.23, 0.76) | 0.004 |
| Death/cardiac death | 10 | 0.95 (0.61, 1.48) | 0.825 | 0.95 (0.61, 1.48) | 0.825 | 8 | 0.39 (0.16, 0.94) | 0.036 | 0.39 (0.16, 0.94) | 0.036 |
| MI | 8 | 0.80 (0.49, 1.32) | 0.387 | 0.80 (0.49, 1.32) | 0.387 | 8 | 0.31 (0.13, 0.74) | 0.008 | 0.31 (0.13, 0.74) | 0.008 |
| **Angiographic Endpoints** |  |  |  |  |  |  |  |  |  |  |
| LLL | 8 | -0.29 (-0.53, -0.04) | 0.021 | -0.26 (-0.39, -0.14) | <0.001 | 6 | -0.75 (-1.02, -0.47) | <0.001 | -0.65 (-0.80, -0.51) | <0.001 |
| Binary restenosis | 6 | 1.06 (0.74, 1.51) | 0.748 | 1.06 (0.74, 1.51) | 0.748 | 5 | 0.31 (0.22, 0.45) | <0.001 | 0.31 (0.22, 0.45) | <0.001 |
| MLD | 10 | -0.48 (-0.67, -0.29) | <0.001 | -0.45 (-0.57, -0.34) | <0.001 | 6 | 0.54 (0.31, 0.76) | <0.001 | 0.50 (0.35, 0.64) | <0.001 |
| DS% | 7 | 0.24 (0.09, 0.40) | 0.001 | 0.24 (0.11, 0.37) | <0.001 | 6 | -0.67 (-0.89, -0.44) | <0.001 | -0.63 (-0.78, -0.49) | <0.001 |

Abbreviations: RE, random effects; FE, fixed effects.

**Supplementary Figure 1. Forest plot of risk ratios for target lesion revascularization associated with DCB vs. the control treatment**

**
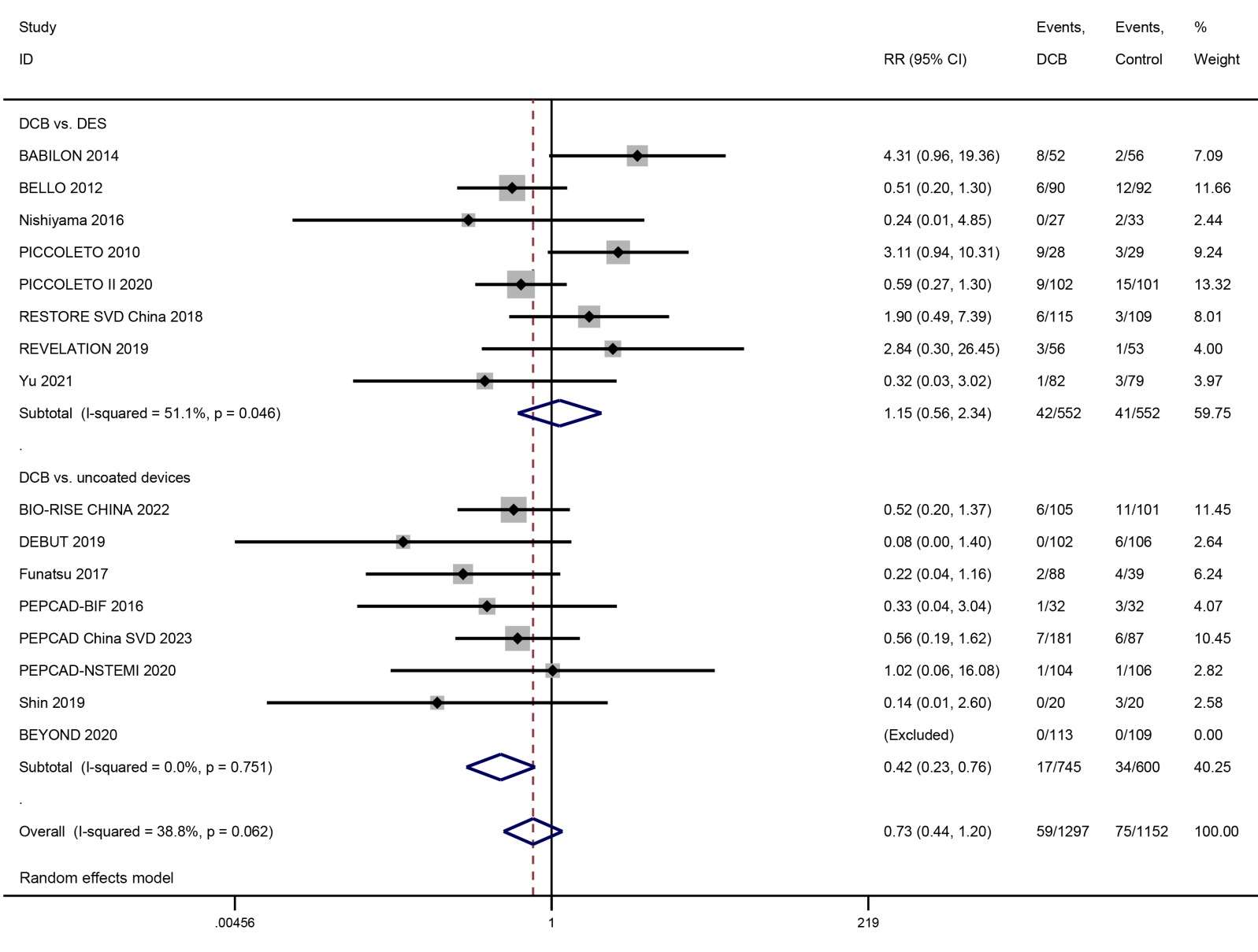
**

Abbreviations: CI, Confidence Interval; DCB, Drug-Coated Balloon; DES, Drug-Eluting Stent; RR, Risk Ratio.

**Supplementary Figure 2. Forest plot of risk ratios for death or cardiac death associated with DCB vs. the control treatment**

**
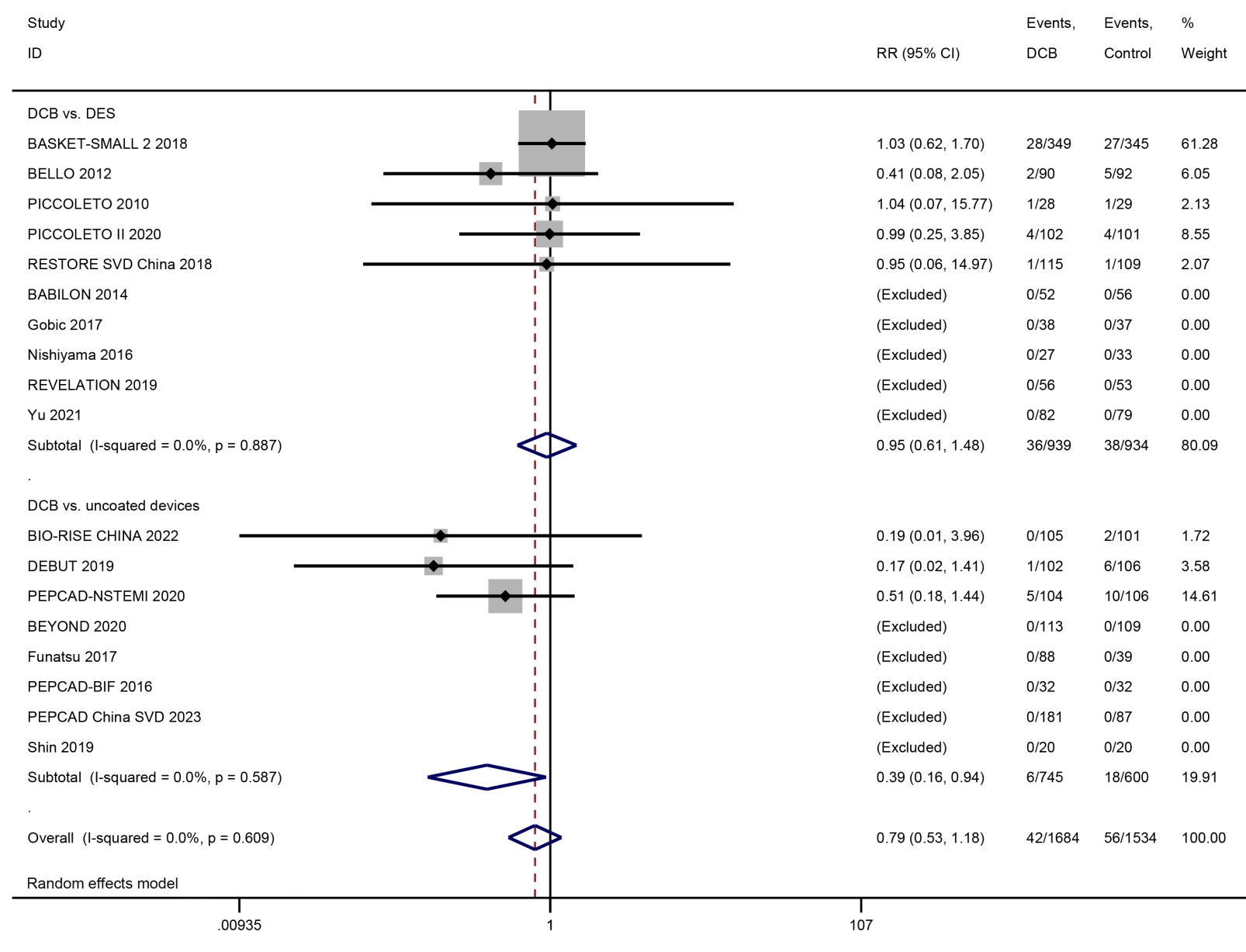
**

Abbreviations: CI, Confidence Interval; DCB, Drug-Coated Balloon; DES, Drug-Eluting Stent; RR, Risk Ratio.

**Supplementary Figure 3. Forest plot of risk ratios for myocardial infarction associated with DCB vs. the control treatment**

**
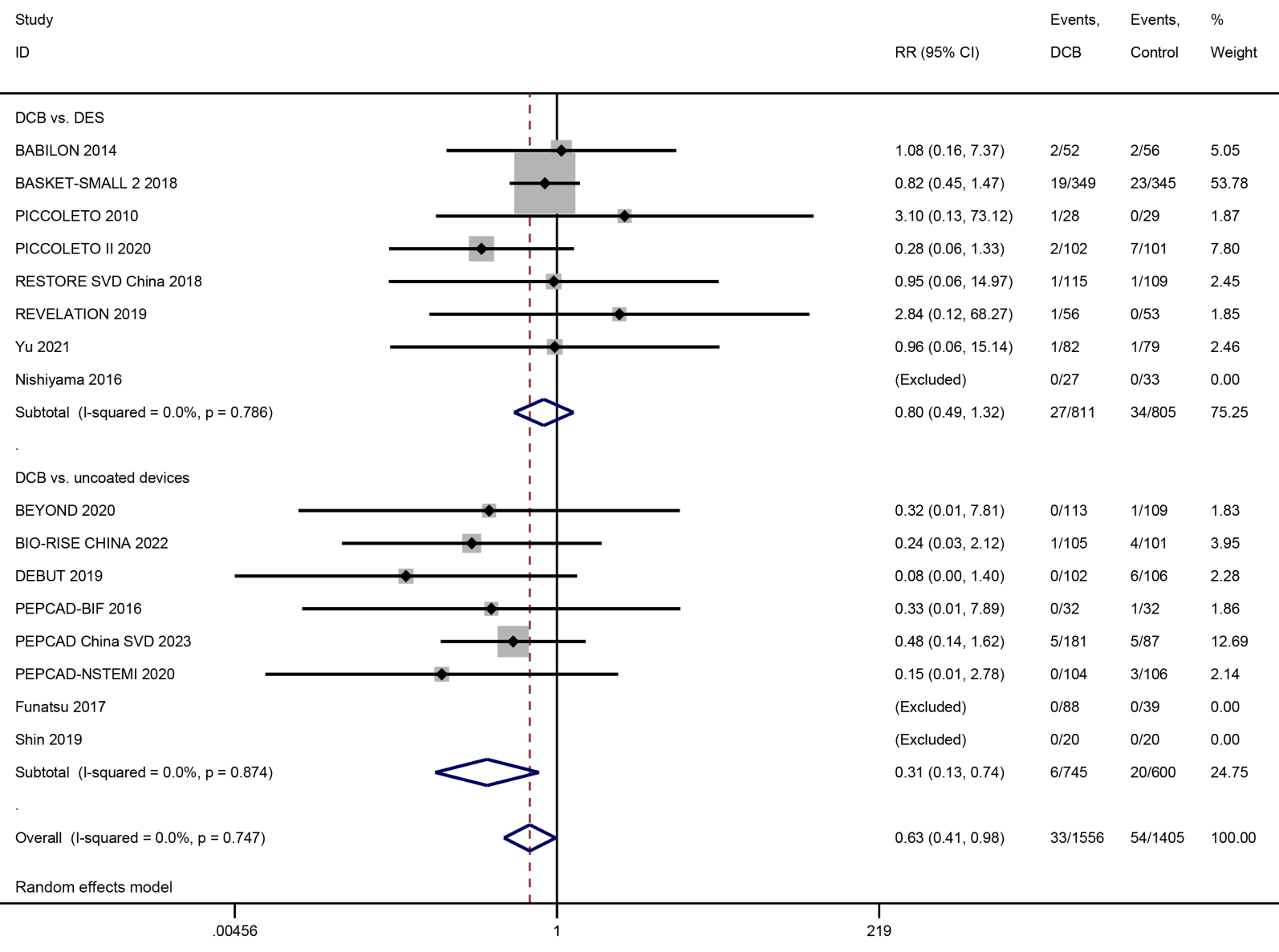
**Abbreviations: CI, Confidence Interval; DCB, Drug-Coated Balloon; DES, Drug-Eluting Stent; RR, Risk Ratio.

**Supplementary Figure 4. Forest plot of risk ratios for binary restenosis associated with DCB vs. the control treatment**

**
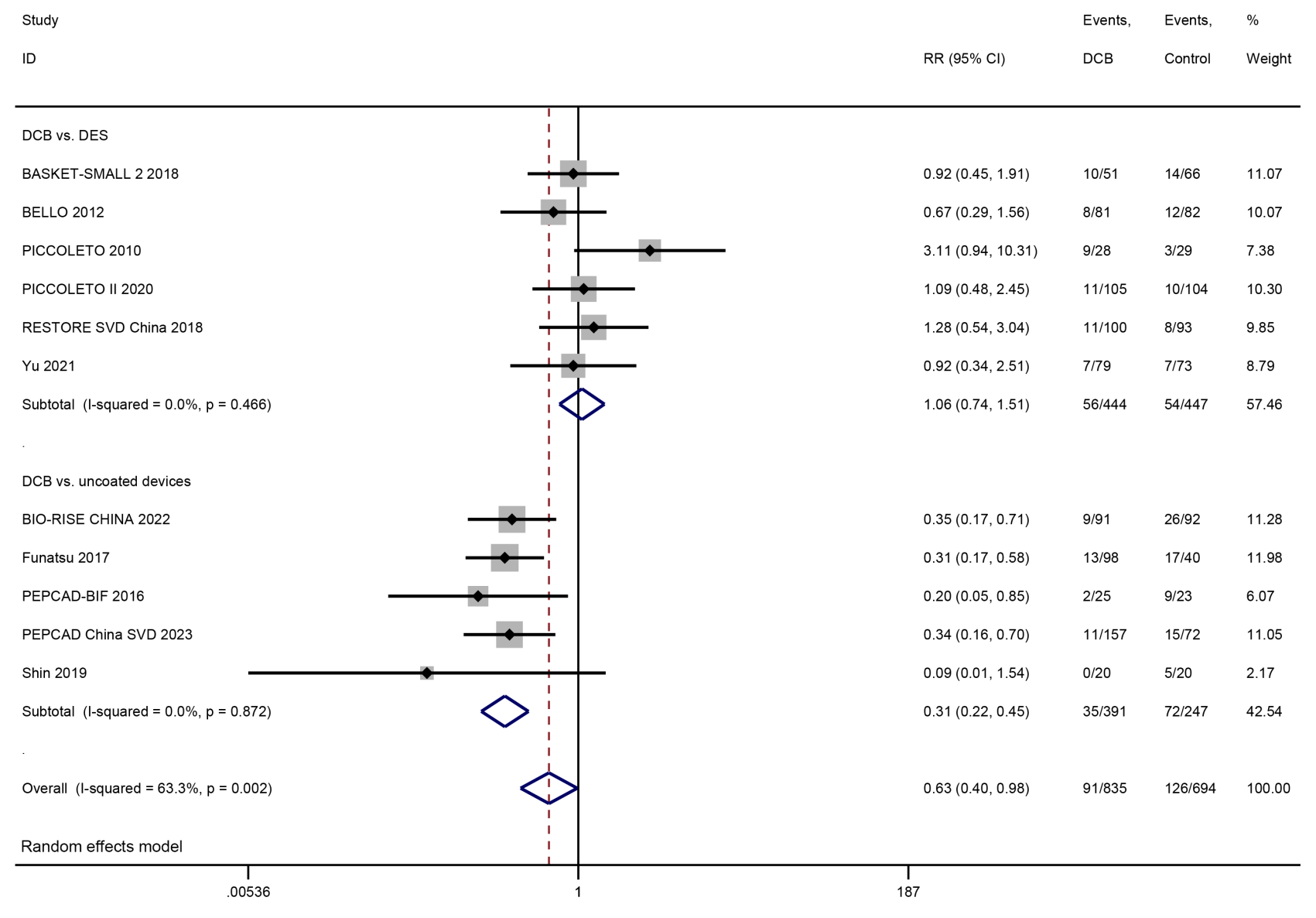
**Abbreviations: CI, Confidence Interval; DCB, Drug-Coated Balloon; DES, Drug-Eluting Stent; RR, Risk Ratio.

**Supplementary Figure 5. Forest plot of mean differences for minimal lumen diameter associated with DCB vs. the control treatment**

**
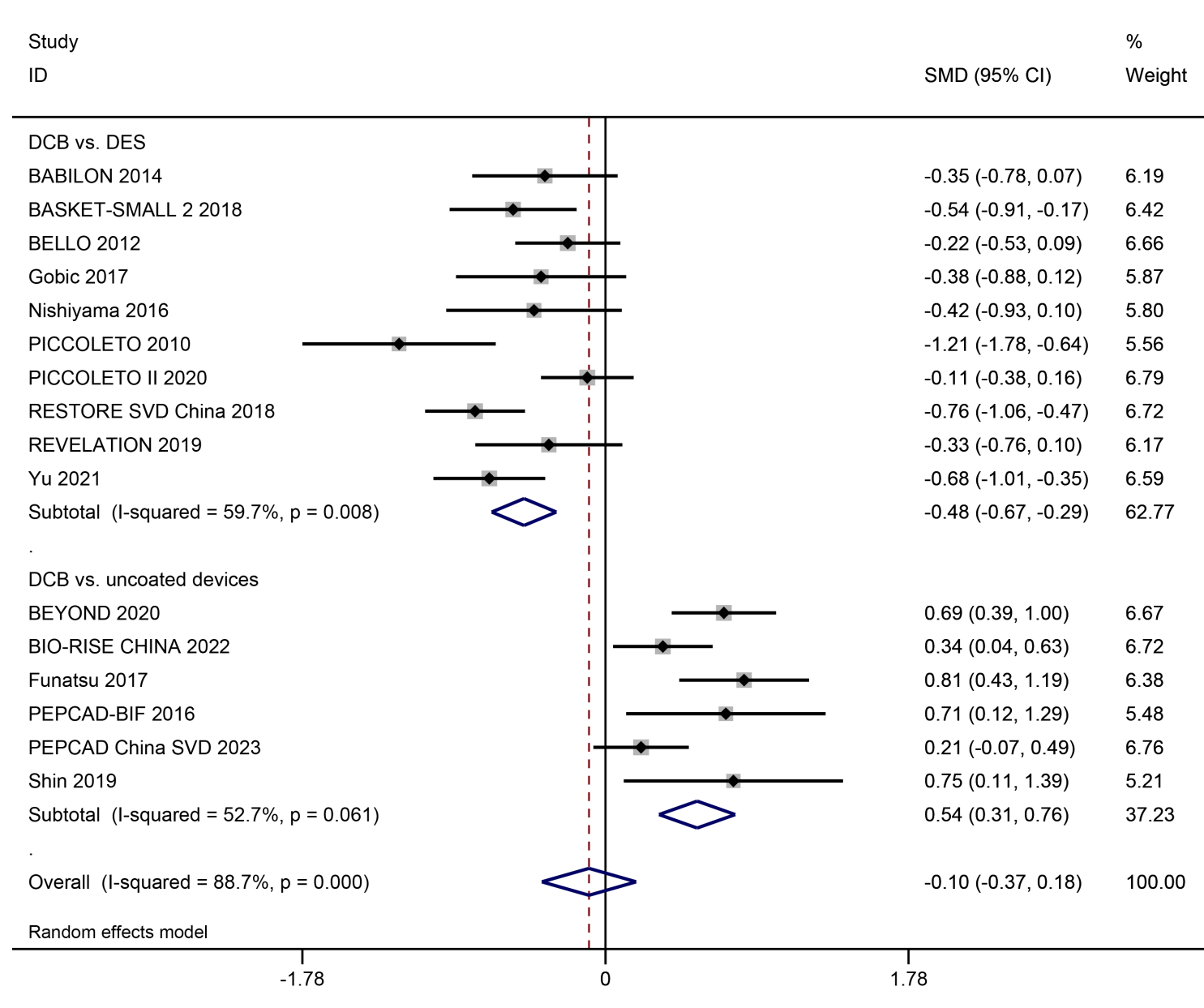
**

Abbreviations: CI, Confidence Interval; DCB, Drug-Coated Balloon; DES, Drug-Eluting Stent; SMD, Standardized Mean Difference.

**Supplementary Figure 6. Forest plot of mean differences for** **percentage diameter stenosis associated with DCB vs. the control treatment**

**
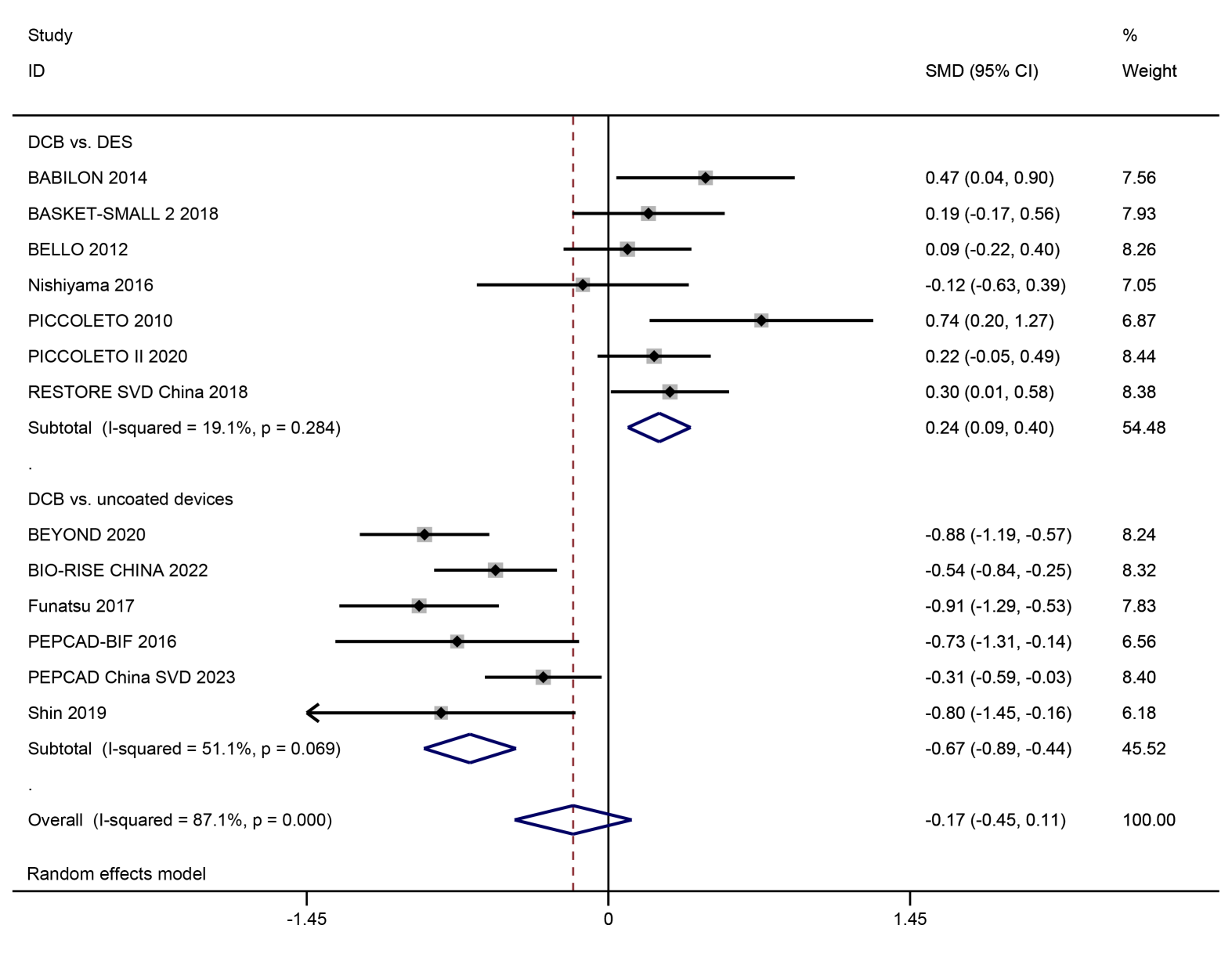
**

Abbreviations: CI, Confidence Interval; DCB, Drug-Coated Balloon; DES, Drug-Eluting Stent; SMD, Standardized Mean Difference.

**Supplementary Figure 7. Bias assessment of the included studies according to the Cochrane Collaboration**

**
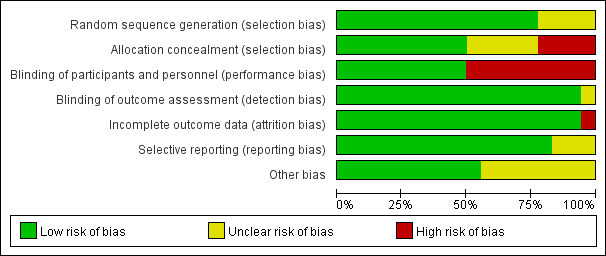
**

**
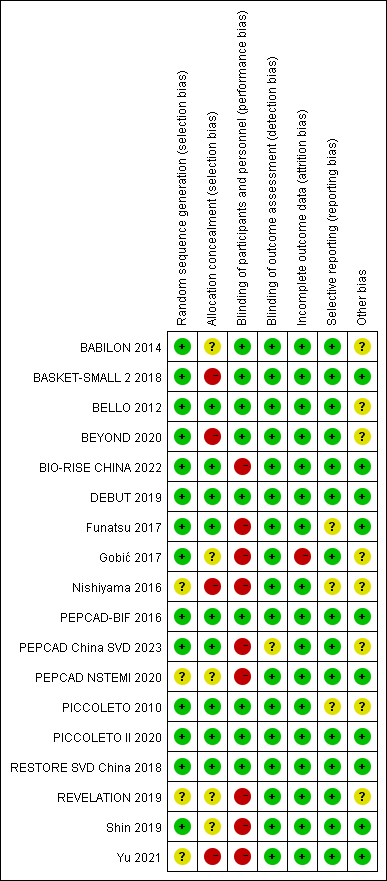
**

**Supplementary Figure 8. Funnel plot for publication bias** **of studies included in the meta-analysis**

**
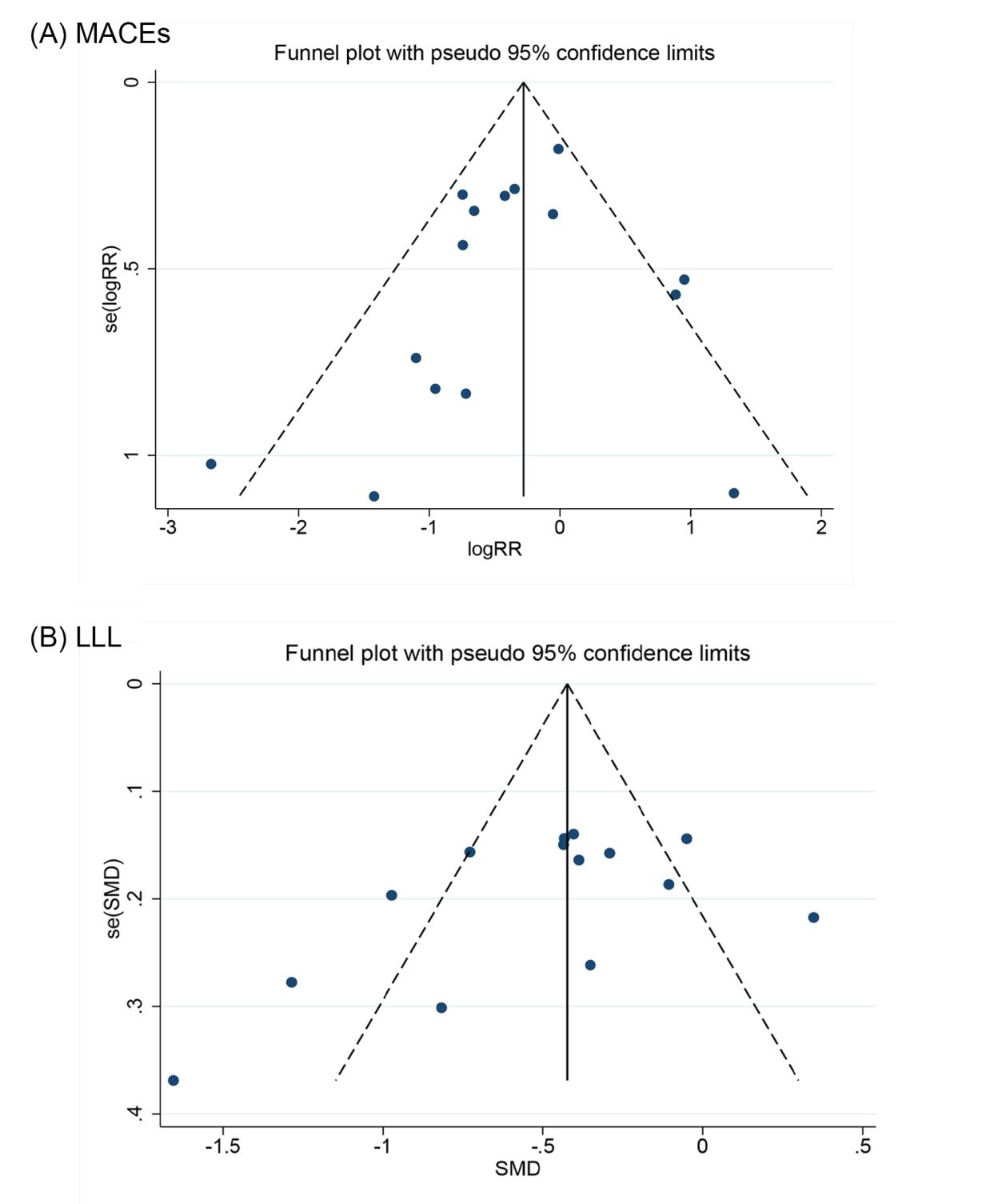
**

Abbreviations: LLL, Late Lumen Loss; MACEs, Major Adverse Cardiovascular Events; SMD, Standardized Mean Difference.

**Supplementary Figure 9. Sensitivity analysis**

**
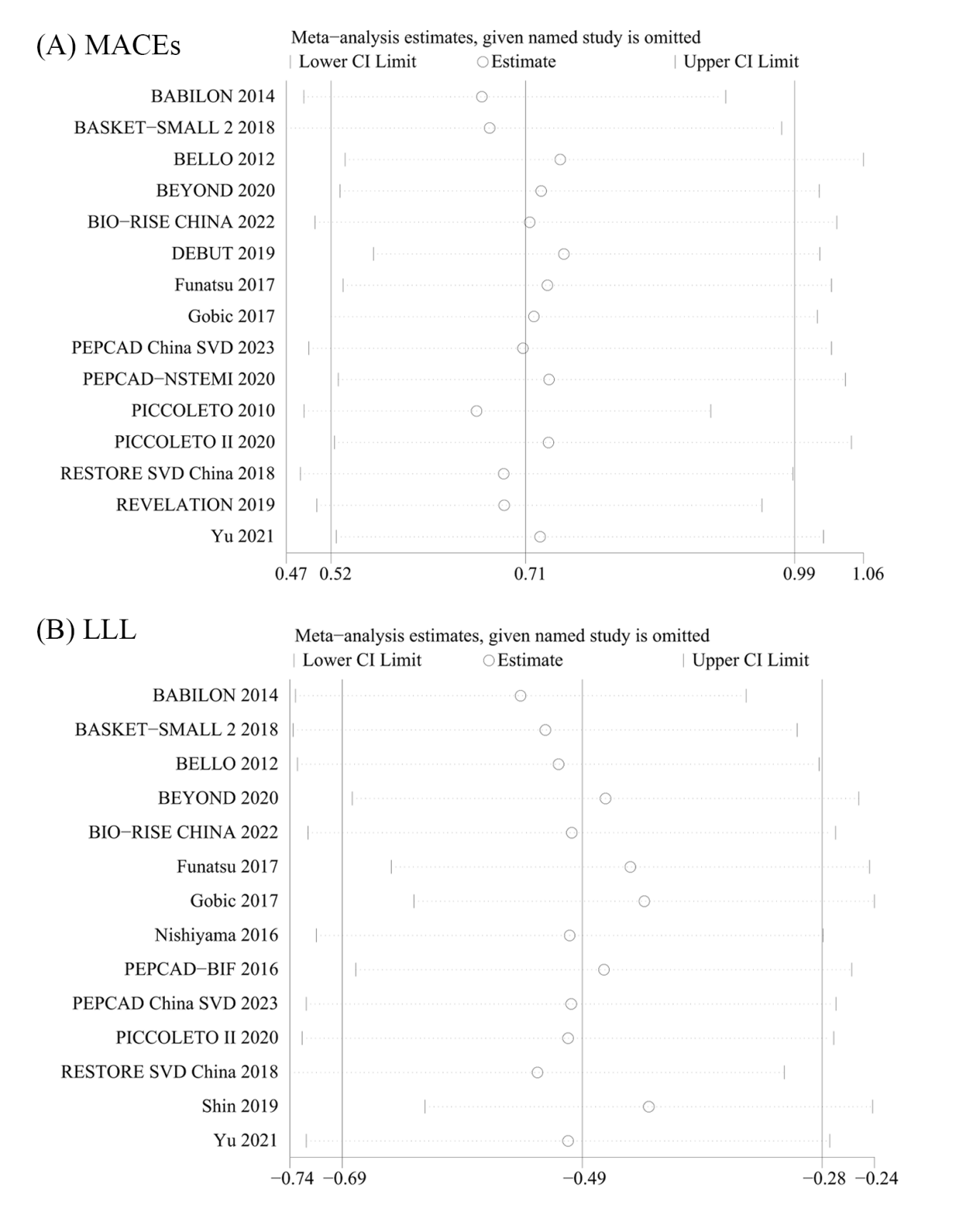
**

Abbreviations: CI, Confidence Interval; LLL, Late Lumen Loss; MACEs, Major Adverse Cardiovascular Events.
